# Supplementary material for: Erythroid Differentiation Regulator 1 Strengthens TCR Signaling by Enhancing PLCγ1 Signal Transduction Pathway
Source: Int J Mol Sci. 2022 Jan 13;23(2):844. doi: 10.3390/ijms23020844 (PMC8776247; doi:10.3390/ijms23020844)
Supplement: Supplementary file 1 [file ijms-23-00844-s001.zip › ijms-1516034-supplementary.pdf]

Supplementary Figures

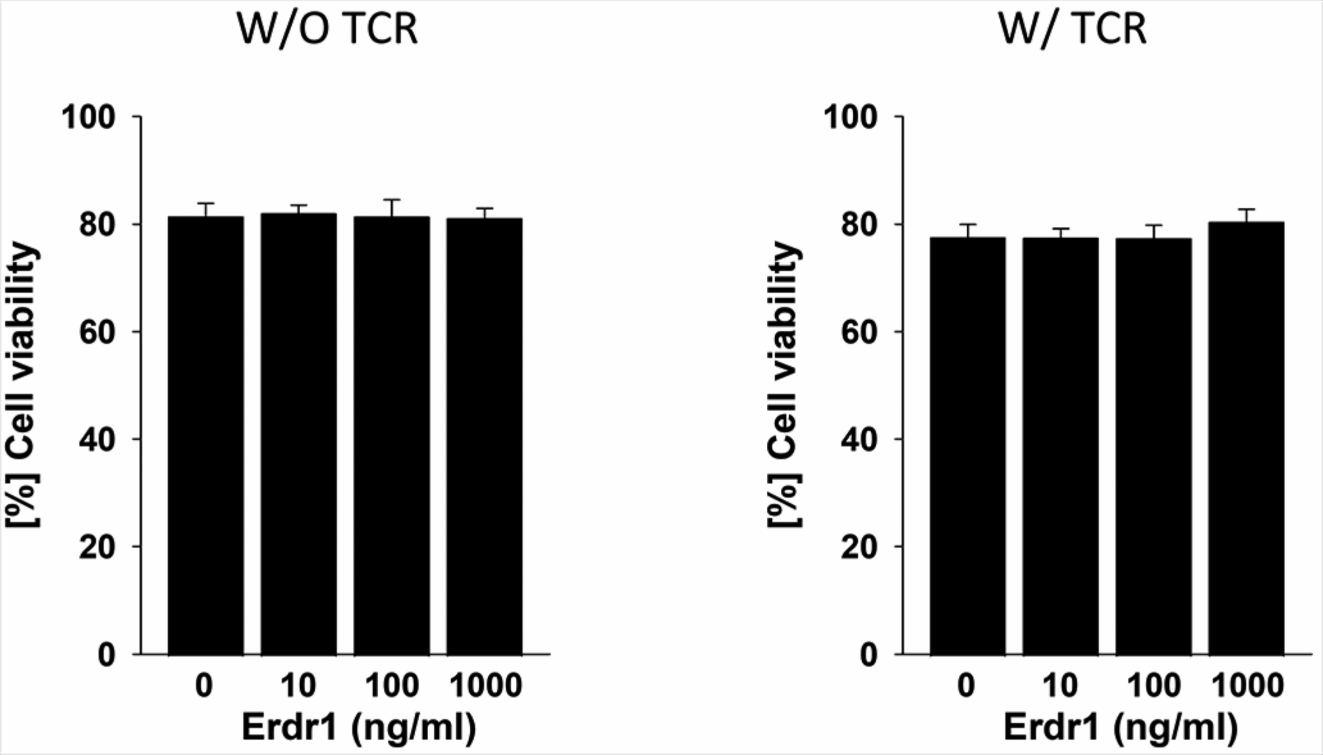

Supplementary Figure S1. Confirmation of the cell viability from flow cytometric analysis.

CD4 T cells from peripheral lymph nodes were cultivated with the indicated concentrations of Erdr1 in the absence or presence of TCR stimulation for 18 h. CD4 T cells were cultured in anti-CD3ε antibody coated plates (0 or 125 ng/ml) and viability was evaluated by flow cytometry. Results were summarized as mean ± SD from three independent experiments.

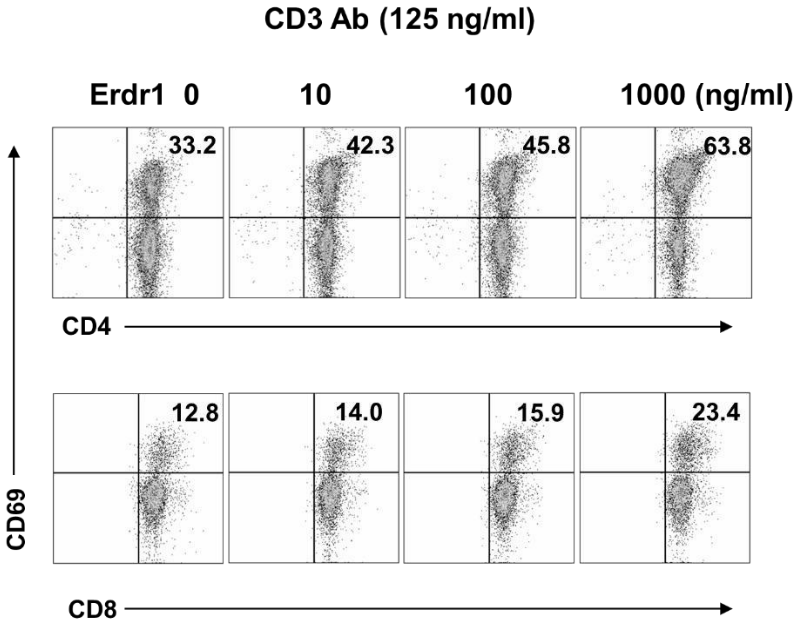

Supplementary Figure S2. Comparison of CD69 expression in CD4 and CD8 T cells with the same concentration of anti-CD3 antibody.

CD4 and CD8 T cells from peripheral lymph nodes were cultivated with the indicated concentrations of Erdr1 in the presence of the anti-CD3 antibody (125 ng/ml). After 18 h, CD69 expression on T cells were determined by flow cytometry. The results of CD4 T cells are from Figure 1.
